# Asn‐linked N–acetylglucosamine of the amylin receptor 2 extracellular domain enhances peptide ligand affinity

**DOI:** 10.1002/2211-5463.13042

**Published:** 2020-12-02

**Authors:** Sangmin Lee

**Affiliations:** ^1^ Department of Basic Pharmaceutical Sciences Fred Wilson School of Pharmacy High Point University High Point NC USA

**Keywords:** amylin receptors, Asn‐linked *N*‐acetylglucosamine, fluorescence polarization, peptide hormones

## Abstract

The calcitonin receptor (CTR) has a large extracellular domain (ECD) with multiple *N*‐glycosylation sites. An asparagine (Asn)‐linked *N*‐acetylglucosamine (GlcNAc) of CTR ECD N130 was previously reported to enhance peptide hormone binding affinity for CTR ECD. CTR forms a complex with an accessory protein RAMP, and the RAMP:CTR complex gains affinity for peptide hormone amylin as the amylin receptor (AMY). Although *N*‐glycosylation of AMY ECD was reported to enhance peptide hormone affinity, it remains underexplored which *N*‐glycosites of AMY ECD are responsible for peptide affinity enhancement and it is unclear whether an Asn‐linked GlcNAc of the *N*‐glycosites plays a critical role. Here, I investigated the role of the Asn‐linked GlcNAc of CTR N130 in the affinity of an antagonistic amylin analog (AC413) for AMY_2_ ECD (the RAMP2 ECD:CTR ECD complex). I used Endo H‐treated CTR ECD in which *N*‐glycans were trimmed to an Asn‐linked GlcNAc on each of the *N*‐glycosites. I incubated Endo H‐treated CTR ECD with excess of glycan‐free RAMP2 ECD to produce the RAMP2 ECD:CTR ECD complex. Using this coincubation system, I found that the RAMP2 ECD complex with Endo H‐treated CTR ECD with N130D mutation showed a fourfold decrease in AC413 affinity compared with the RAMP2 ECD complex with Endo H‐treated CTR ECD WT. In contrast, RAMP2 ECD *N*‐glycosylation did not affect peptide binding affinity. These results indicate that the Asn‐linked GlcNAc of CTR N130 is an important peptide affinity enhancer for AMY_2_ ECD and reveals a significant role of the Asn‐linked GlcNAc in AMY_2_ function.

## Graphical abstract

The amylin receptor 2 is the complex of the calcitonin receptor and an accessary protein called receptor activity‐modifying protein 2. The current study shows that the asparagine (Asn)‐linked *N*‐acetylglucosamine (GlcNAc) of the calcitonin receptor significantly enhances peptide ligand affinity for the amylin receptor 2 extracellular domain.

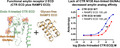

**Abbreviations**
AM: adrenomedullin
AMY: amylin receptor
Asn: asparagine
CGRP: calcitonin gene‐related peptide
CLR: calcitonin receptor‐like receptor
CTR: calcitonin receptor
GlcNAc: *N*‐acetylglucosamine
Man: mannose
RAMP: receptor activity‐modifying protein
sCT: salmon calcitonin

*N*‐glycosylation is an important post‐translational modification for expression, proper folding, and function of proteins [1]. G protein‐coupled receptors (GPCR) are cell surface proteins that interact with various extracellular signaling molecules, and their activation mediates multiple physiological processes [2]. *N*‐glycosylation of a subset of GPCRs is reported critical for their membrane expression, ligand interaction, and cell signaling activation [3, 4, 5]. Extracellular domains (ECDs) or loops of GPCRs are known as the sites for *N*‐glycosylation, and in particular, class B GPCRs activated by various peptide hormones are characterized by a large ECD with multiple *N*‐glycosylation sites.

The calcitonin receptor (CTR) is a class B GPCR for peptide hormone calcitonin that regulates calcium homeostasis. CTR has an ECD with four *N*‐glycosylation sites: N28, N73, N125, and N130. N28 was reported to be absent in a functional splice variant of CTR indicating that *N*‐glycosylation of N28 is dispensable for CTR function [6]. Recent reports showed that *N*‐glycosylation of CTR significantly contributes to peptide hormone binding affinity [4, 7, 8]. My colleagues and I reported that *N*‐glycosylation of CTR enhanced salmon calcitonin (sCT) affinity for CTR ECD by sevenfold [4] and that the asparagine (Asn)‐linked *N*‐acetylglucosamine (GlcNAc) residue of CTR N130 is mostly responsible for this effect [4, 8]. sCT affinity for full‐length CTR was also enhanced by the *N*‐glycosylation of CTR ECD N130 [7]. *N*‐glycosylation of CTR ECD N73 and N125 showed a minimum or moderate effect on peptide affinity enhancement, suggesting that N130 is the most responsible site for the *N*‐glycosylation effect [4].

Receptor activity‐modifying proteins (RAMPs) are the accessory proteins with one transmembrane domain and an ECD. RAMPs are known to interact with GPCRs to modulate their function [9]. RAMPs were initially reported with their effects on the CTR‐like receptor (CLR) [10]. RAMPs have three types in humans, and their heterodimer complexes with CLR are the calcitonin gene‐related peptide (CGRP) receptor or the adrenomedullin (AM) receptor depending on the interacting RAMP; the RAMP1:CLR complex is the receptor for CGRP, and the RAMP2:CLR complex is the receptor for AM (AM_1_ receptor) [10]. RAMPs can form a heterodimer complex with CTR, and the RAMP:CTR complexes gain affinity for peptide hormone amylin [11, 12, 13]. All three types of RAMP:CTR complexes are the amylin receptors (AMY), which are presented as RAMP1:CTR (AMY_1_), RAMP2:CTR (AMY_2_), and RAMP3:CTR (AMY_3_). AMY activation controls blood glucose and food intake, and a peptide drug targeting AMY is used to treat diabetes as conjunction therapy with insulin [14].

Previous reports showed that *N*‐glycosylation enhances peptide ligand affinity for AMY [4, 8]. My colleagues and I established a RAMP2 ECD and CTR ECD fusion protein (RAMP2‐CTR ECD fusion) and found that the glycan‐free RAMP2‐CTR ECD fusion protein produced from *Escherichia coli* showed a fivefold decrease in an antagonistic amylin analog AC413 affinity compared with the fully glycosylated version [4]. When the RAMP2‐CTR ECD fusion protein was enzymatically deglycosylated by PNGase F, AC413 affinity was decreased by over 10‐fold [4, 8]. In addition, we constructed a RAMP1 ECD and CTR ECD fusion (RAMP1‐CTR ECD fusion) and showed that PNGase F‐catalyzed deglycosylation decreased AC413 affinity for RAMP1‐CTR ECD fusion by over 10‐fold [8]. We found that *N*‐glycosylation of CTR N130 is mostly responsible for the affinity decrease at least for the RAMP1‐CTR ECD fusion protein (AMY_1_ ECD) [8]. However, whether the CTR N130 *N*‐glycosite also plays a critical role in AMY_2_ ECD and to what level the Asn‐linked GlcNAc contributes to the *N*‐glycosylation effect on AMY_2_ ECD still have not been addressed.

Here, I developed a RAMP2 ECD and CTR ECD coincubation system where the functional RAMP2 ECD:CTR ECD complex formation was enforced. Although human RAMP2 ECD contains one *N*‐glycosylation site, functional glycan‐free RAMP2 ECD was obtained from *E. coli* in a large quantity [15]. Glycan‐free RAMP2 ECD was coexpressed with disulfide bond isomerase DsbC from *E. coli* and was suggested properly folded [16]. Using the functional glycan‐free RAMP2 ECD coincubation, I examined the role of the Asn‐linked GlcNAc of CTR N130 in the enhancement of peptide ligand affinity for AMY_2_ ECD.

## Materials and methods

### Reagents

Dulbecco's modified Eagle's media (DMEM) with 4.5 g·L^−1^ glucose and l‐glutamine and 100X stock solution of nonessential amino acids (NEAA) were purchased from Lonza (Basel, Switzerland). FBS used for mammalian cell culture was purchased from Life Technologies (Carlsbad, CA, USA). Gibson Assembly Master Mix, restriction enzymes, PNGase F, and Endo H were purchased from New England Biolabs (NEB, Ipswich, MA, USA). All other reagents were obtained from Sigma‐Aldrich (St. Louis, MO, USA), unless otherwise noted.

### Cell line and bacterial cells used

HEK293S GnTI^−^ cells were purchased from ATCC (Manassas, VA, USA). HEK293S GnTI^−^ cells were used to express glycosylated CTR ECD with *N*‐glycans (Man_5_GlcNAc_2_) that can be trimmed to an Asn‐linked GlcNAc by Endo H treatment. HEK293S GnTI^−^ cells were cultured at 37 °C, 5% CO_2_ in DMEM plus 10% FBS with supplemental 1X NEAA. Origami B DE3 cells were used for the expression and purification of glycan‐free CTR ECD and RAMP2 ECD as previously described [8, 15].

### Expression plasmids

pHLsec‐based vectors were used to obtain glycosylated CTR ECD from HEK293S GnTI^−^ cells as a secreted protein. Maltose‐binding protein (MBP) was fused to the CTR ECD to increase initial protein expression and purification. The following constructs used in this study were previously described [8]: pHLsec/MBP‐TEV cleavage site‐hCTR.38‐141‐H_6_ WT (pSL031) and N130D (pSL033). pETDuet1‐based vectors were used for the expression of glycan‐free proteins from *E. coli*. pETDuet1/MBP‐Thrombin cleavage site‐hRAMP2.55‐140‐H_6_ (pHH049) was constructed using a traditional cloning method as previously described [15]. pETDuet1/hRAMP2.55‐140‐H_6_ (H‐pSL010) was also constructed using Gibson Assembly reaction to express RAMP2 ECD without an MBP‐tag. pETDuet1/MBP‐TEV cleavage site‐CTR.38‐141‐H_6_ (pSL035) for glycan‐free CTR ECD expression was constructed as previously described [8]. For the expression of the RAMP2‐CTR ECD fusion protein, the following pHLsec‐based vectors were used: pHLsec/hRAMP2.55‐140‐(GSA)_3_‐hCTR.34‐141‐H_6_ (H‐pSL006) and pHLsec/hRAMP2.55‐140.N130D‐(GSA)_3_‐hCTR.34‐141‐H_6_ (H‐pSL008). The following pHLsec‐based vectors were used to express MBP‐tagged RAMP2‐CLR ECD fusion proteins: pHLsec/MBP‐hRAMP2.55‐140‐(GS)_5_‐hCLR.29‐144‐H_6_ (pSL013) and pHLsec/MBP‐hRAMP2.55‐140.N130D‐(GS)_5_‐hCLR.29‐144‐H_6_ (pSL027).

### Expression and purification of glycosylated CTR ECD WT and N130D

MBP‐TEV cleavage site‐CTR ECD WT was expressed from HEK293S GnTI^−^ cells for 3 days after plasmid transfection with polyethyleneimine (PEI). All protein purification steps were performed at 4 °C, otherwise noted. The expressed protein was purified with immobilized Ni metal affinity chromatography (IMAC). The peak fractions were pooled and incubated with 1 : 5 ratio TEV protease (w/w, TEV protease: protein) for 6 h at 20 °C. The reaction mixture was spin‐concentrated (MWCO 10 kDa) and loaded to size‐exclusion chromatography (SEC). The fractions with expected CTR ECD WT molecular weight were collected, spin‐concentrated, and used for PNGase F reaction and following peptide binding assay. MBP‐TEV cleavage site‐CTR ECD N130D was expressed and purified as described above except an additional purification step to achieve higher purity. After TEV protease reaction, the reaction mixture including CTR ECD N130D was loaded to the second IMAC to remove free and untagged MBP. The peak fractions were spin‐concentrated and loaded to SEC. After SEC, the peak fractions of CTR ECD N130D were spin‐concentrated and used for PNGase F reaction and peptide binding assay.

### Expression and purification of Endo H‐treated CTR ECD WT and N130D

The expression and purification procedure of Endo H‐treated CTR ECD WT and N130D was previously reported [8]. Briefly, MBP‐TEV cleavage site‐CTR ECD WT and N130D were expressed from HEK293S GnTI^−^ cells. The expressed protein was purified with IMAC, and the peak fractions were incubated with 1 : 5 ratio TEV protease (w/w, TEV protease : protein) for 6 h at 20 °C. After TEV protease reaction, *N*‐glycans of the protein were trimmed with 1 : 10 ratio Endo H (w/w, Endo H : protein) overnight at 4 °C, while dialyzing to 25 mm MOPS (pH 6.5) and 150 mm NaCl. Next day, the pH was increased to pH 7.5 and the protein was purified with IMAC and following SEC. The final peak fractions were dialyzed to storage buffer and stored at −80 °C until use.

### Glycan‐free RAMP2 ECD and CTR ECD expression and purification

The expression and purification of glycan‐free RAMP2 ECD were previously reported except for using RAMP2.55‐140 instead of RAMP2.36‐140 [15]. Briefly, MBP‐Thrombin cleavage site‐hRAMP2.55‐140‐H_6_ was expressed in the *E. coli* strain Origami B DE3. The cells were lysed by sonication and spun down by centrifugation. The supernatant was purified with IMAC and following amylose affinity chromatography. Human α‐thrombin was used to cleave out MBP, and the reaction mixture was loaded to IMAC to remove untagged MBP and thrombin. The RAMP2 ECD was further purified with SEC, and the peak fractions were dialyzed to storage buffer and stored at −80 °C until use. RAMP2 ECD without an MBP‐tag was also purified as follows. hRAMP2.55‐140‐H_6_ was expressed in the *E. coli* strain Origami B DE3. The bacterial cells were lysed by sonication using Bioruptor® standard (Diagenode Inc., Denville, NJ, USA). The cells were centrifuged, and the supernatant was purified with IMAC and following SEC. The peak fractions from SEC were dialyzed to storage buffer and stored at −80 °C until use.

Glycan‐free CTR ECD expression and purification were previously reported [8]. MBP‐TEV cleavage site‐CTR ECD was expressed in the *E. coli* strain Origami B DE3. The expressed protein was purified with IMAC, and the peak fractions were incubated with *E. coli*‐produced RAMP2 ECD overnight at 20 °C to facilitate proper folding. Next day, the reaction mixture was purified with amylose affinity chromatography and following SEC. The peak fractions were incubated with 1 : 5 ratio TEV protease (w/w, TEV protease : protein) for 6 h at 20 °C. The reaction mixture was proceeded to dialysis to remove bound maltose, following amylose affinity chromatography, and IMAC. The final peak fractions were dialyzed to storage buffer and stored at −80 °C until use.

### RAMP2‐CTR ECD fusion protein and MBP‐RAMP2‐CLR ECD fusion protein purification

RAMP2 WT‐CTR ECD fusion and RAMP2[N130D]‐CTR ECD fusion proteins were expressed in HEK293T cells at 37˚C for 4 days after plasmid transfection with PEI. The expressed protein was purified with IMAC, and the peak fractions were collected and purified with following SEC. The final fractions were dialyzed to storage buffer overnight, and stored at −80 °C until use. For MBP‐RAMP2 WT‐CLR ECD fusion and MBP‐RAMP2[N130D]‐CLR ECD fusion proteins, the purification procedures were the same as those of the RAMP2‐CTR ECD fusion proteins except being expressed from HEK293S GnTI^−^ cells for 3 or 4 days after transfection with PEI.

### Synthetic peptides

Fluorescein isothiocyanate (FITC)‐labeled peptide probes were custom‐synthesized and HPLC‐purified by RS Synthesis (Louisville, KY, USA) or Genscript (Piscataway, NJ, USA). FITC extinction coefficient (63 000 m ^−1^·cm^−1^ at 495 nm, pH 7.0) was used to measure concentrations of FITC‐labeled peptides. The following peptides were used in the current study: FITC‐Ahx (aminohexanoic acid)‐sCT(22–32), FITC‐Ahx‐YPRTNTGSGTP‐NH_2_; FITC‐Ahx‐AC413(6–25), FITC‐Ahx‐ANFLVRLQTYPRTNVGANTY‐NH_2_; FITC‐Ahx‐AM(37–52)[S45W/Q50W], FITC‐Ahx‐DKDNVAPRWKISPWGY‐NH_2_.

### Fluorescence polarization/anisotropy peptide binding assay

The procedure of fluorescence polarization (FP) peptide binding assay was previously described [4]. FITC‐sCT(22–32), FITC‐AC413(6–25), and FITC‐AM(37–52)[S45W/Q50W] were used as fluorescence‐labeled peptide probes. The reaction buffer used in peptide binding assay was 50 mm HEPES (pH 7.4), 150 mm NaCl, 0.5 mg·mL^−1^ fatty acid‐free BSA, 0.5 mm maltose, 0.5 mm EDTA, and 0.1% (v/v) Tween‐20. The procedure of the PNGase F treatment and following FP peptide binding assay was previously described [4]. Briefly, 4 μL of PNGase F (500 U·μL^−1^) was used to deglycosylate 10 μg of CTR ECD WT or 30 μg of CTR ECD N130D in the reaction volume of 44 μL for 4 h at 37 °C. After PNGase F treatment, the reaction mixture was directly used for FP peptide binding assay and the deglycosylation was confirmed with SDS/PAGE. For RAMP2 ECD coincubation, purified RAMP2 ECD was initially diluted with the reaction buffer, concentrated with spin concentrators (MWCO 3 kDa or 10 kDa; Milliporesigma, Burlington, MA, USA), and then added to the reaction mixture as a final concentration 100 μm. A PolarStar Omega plate reader (BMG Labtech, Cary, NC, USA) or a SpectraiD5 (Molecular Devices, San Jose, CA, USA) was used to measure FP. Background‐subtracted fluorescence intensity was used to calculate anisotropy. G factor was used to correct instrumental bias for parallel or perpendicular fluorescence intensity for the SpectraiD5. G factor was determined as 0.42 where the polarization (mP) of a free‐state FITC probe produces 50 mP. Anisotropy calculated from FP was corrected when total fluorescent intensity changes over 10% as previously reported [4]. The corrected anisotropy was used for nonlinear regression curve fitting using prism 5.0 (GraphPad Software, San Diego, CA, USA). For the appropriate curve fitting, the maximal anisotropy values were shared among the groups shown in Fig. 4A,B. Maximal anisotropy values were not constrained between the groups in other Figures.

### Statistical analysis

A one‐way ANOVA with three groups was performed with prism5.0, and Tukey's *post hoc* test was followed for multiple comparisons. prism5.0 was also used to perform a two‐tailed Student's *t*‐test when two groups were compared. *P* < 0.05 was regarded as statistical significance.

## Results

### Confirmation of the *N*‐glycosylation effect of CTR ECD on peptide hormone affinity

**Fig. 1:**
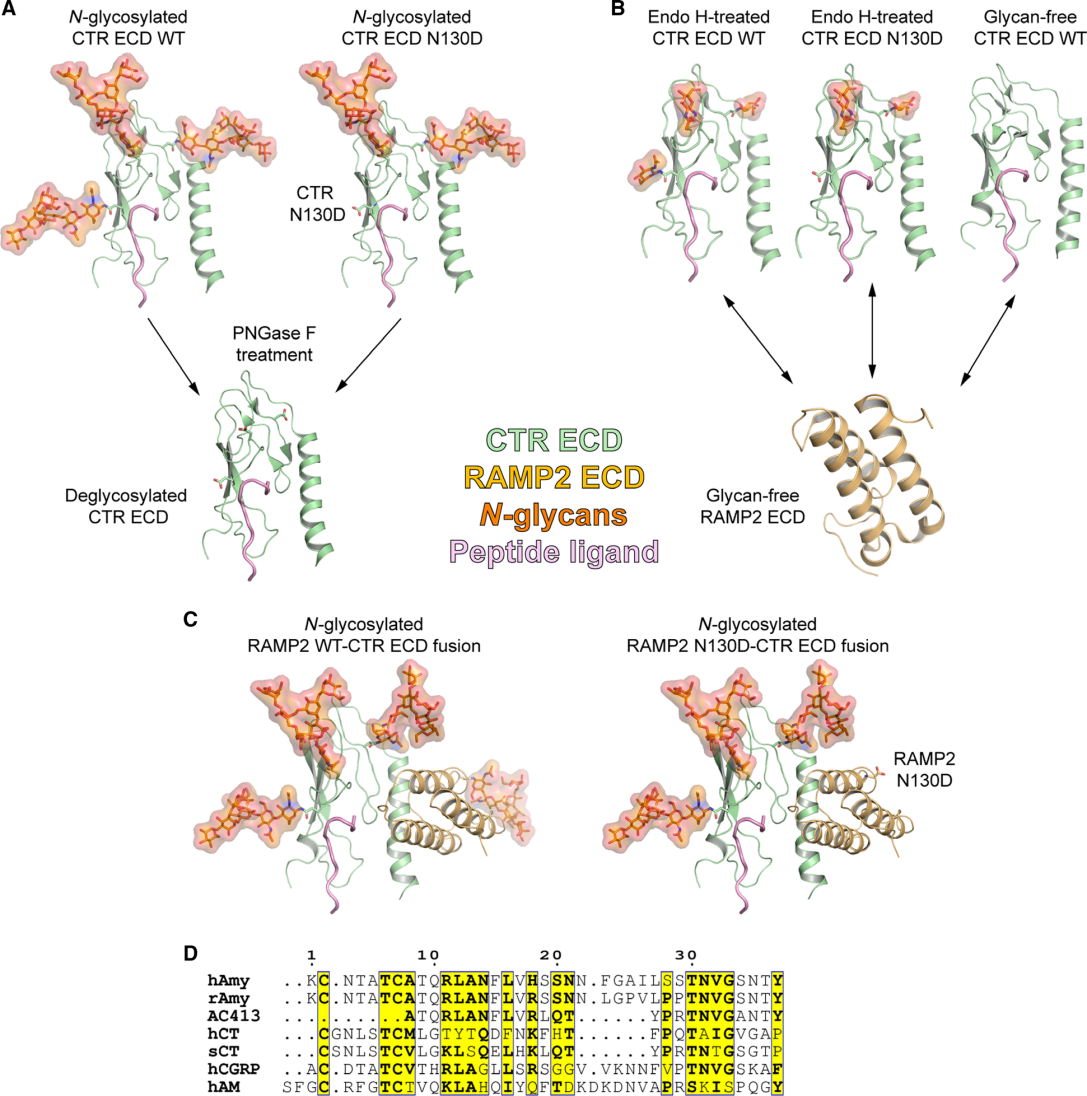
*N*‐glycosylated receptor ECDs and peptide ligands used in this study. (A) *N*‐glycosylated CTR ECD WT and CTR ECD with the N130D mutation. These proteins were expressed HEK293S GnTI^−^ cells and were used for PNGase F experiments and following peptide binding assay in Fig. 2. The crystal structure of glycosylated CTR ECD with sCT (PDB: 6PGQ) was used, and *N*‐glycans of Man_5_GlcNAc_2_ were added by glyprot [17]. The N130D mutation of CTR ECD was introduced by using mutagenesis in pymol(Schrödinger, New York, NY, USA). Deglycosylated CTR ECD by PNGase F had Asn‐to‐Asp conversion by the enzyme, and this conversion was introduced by using mutagenesis in pymol. (B) Endo H‐treated/Asn‐linked GlcNAc‐containing CTR ECD WT (PDB: 6PGQ) and glycan‐free CTR ECD (PDB: 5II0). The N130D mutation of CTR ECD was introduced by using mutagenesis in pymol. Glycan‐free RAMP2 ECD was from the crystal structure of RAMP2‐CLR ECD fusion (PDB: 4RWF). These receptor ECDs were used for Figs 3 and 4. (C) *N*‐glycosylated RAMP2 WT or N130D‐CTR ECD fusion proteins. The structural model of the RAMP2 ECD:CTR ECD complex was produced using crystal structures of RAMP2‐CLR ECD (PDB: 4RWF) and CTR ECD (PDB: 6PGQ). CTR ECD was aligned with CLR ECD of RAMP2‐CLR ECD, and CLR ECD was removed to obtain the RAMP2 ECD:CTR ECD complex. *N*‐glycans of Man_5_GlcNAc_2_ were added by glyprot [17]. *N*‐glycans of Man_5_GlcNAc_2_ were shown for brevity instead of complex *N*‐glycans that were added to the RAMP2‐CTR ECD fusion proteins produced from HEK293T cells. The N130D mutation of RAMP2 ECD was introduced by using mutagenesis in pymol. These receptor ECDs were used for Fig. 5A. (D) Sequence alignment of peptide ligands related to this study. ClustalX2 was used for sequence alignment, and espript 3.0 (http://espript.ibcp.fr) was used for figure representation. Similar residues were shown in yellow boxes and black bold characters. Human α‐CGRP was used. h, human being; r, rat; s, salmon.

My colleagues and I previously reported that the *N*‐glycosylation of CTR ECD enhances peptide hormone affinity and the *N*‐glycosite of CTR N130 is most responsible for this effect [4, 8]. However, the deglycosylation effect of CTR ECD with the N130D mutation by PNGase F was not tested and this approach provides another measure to confirm the previous report. If CTR N130 *N*‐glycans are important for sCT affinity enhancement as previously reported [4], the CTR N130D mutation and following PNGase F‐catalyzed deglycosylation would not markedly decrease sCT affinity. I expressed and purified glycosylated CTR ECD with the N130D mutation (CTR ECD[N130D]) from HEK293S GnTI^−^ cells where *N*‐glycans of N130 are absent. Then, the CTR ECD[N130D] was enzymatically deglycosylated by PNGase F. The deglycosylated CTR ECD[N130D] was used for FP peptide binding assay to directly measure the binding affinity of FITC‐labeled sCT for CTR ECD as previously described [4]. In Fig. 1, the *N*‐glycosylation status of receptor ECD proteins used in this study was illustrated using glyprot [17] and peptide sequence alignment (Fig. 1D) was shown to help better understanding of this work.

**Fig. 2:**
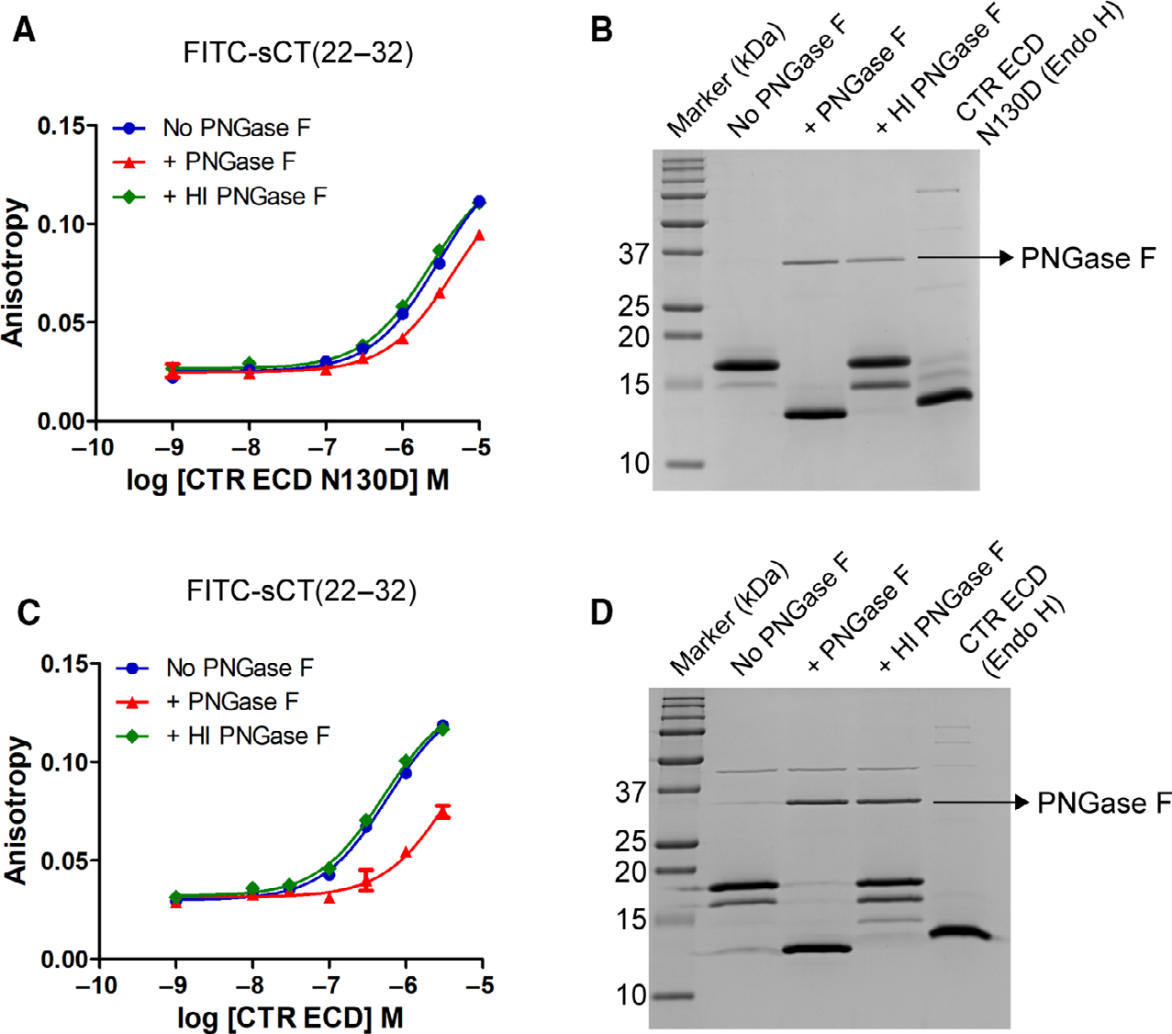
FITC‐sCT(22–32) binding to deglycosylated CTR ECD[N130D] and CTR ECD WT by PNGase F treatment. (A, C) FITC‐sCT(22–32) binding to CTR ECD[N130D] or CTR ECD WT after PNGase F treatment. CTR ECD[N130D] and CTR ECD WT expressed from HEK293S GnTI^−^ cells were deglycosylated with PNGase F for 4 h at 37 °C. Heat‐inactivated (HI) PNGase F was used as a negative control. Duplicate samples per each concentration were used, and SEM was shown as error bars. When the error bars of the duplicate samples were shorter than the height of the symbol in the representative curves, they were omitted. (B, D) SDS/PAGE and following Coomassie blue staining of CTR ECD[N130D] and CTR ECD WT. Samples in a reducing condition with excess of dithiothreitol were loaded. Endo H‐treated CTR ECD[N130D] and CTR ECD WT where *N*‐glycans were trimmed to Asn‐linked GlcNAc were used for molecular weight comparison.

As expected, PNGase F treatment with CTR ECD[N130D] decreased sCT affinity by 1.7‐fold compared with the sCT affinity for No PNGase F treatment (Figs 1A and 2A). Heat‐inactivated PNGase F treatment was used as a negative control, and it did not change sCT affinity for CTR ECD[N130D] (Fig. 2A). This indicates that the presence of inactive PNGase F does not interfere with molecular interaction between CTR ECD and sCT (Fig. 2A). p*K*_D_ (Mean ± SD) of FITC‐sCT(22–32) for glycosylated CTR ECD[N130D] with + PNGase F and with + heat‐inactivated PNGase F was 5.31 ± 0.03 (*K*_D_ 4.90 ± 0.32 μm) and 5.62 ± 0.04 (*K*_D_ 2.43 ± 0.20 μm) from two independent experiments. p*K*_D_ of FITC‐sCT(22–32) for glycosylated CTR ECD[N130D] without any treatment (No PNGase F) was 5.54 (*K*_D_ 2.88 μm; Fig. 2A). Glycosylated CTR ECD[N130D] was fully deglycosylated by PNGase F treatment as shown in SDS/PAGE and following Coomassie blue staining (Fig. 2B). Endo H‐treated CTR ECD N130D that has an Asn‐linked GlcNAc residue on N73 and N125 *N*‐glycosylation sites was used for the molecular weight comparison to the fully deglycosylated CTR ECD N130D by PNGase F (Fig. 2B). These results confirm the previous reports that *N*‐glycans of CTR N130 are mostly responsible for the peptide hormone affinity enhancement for CTR ECD [4, 8].

I also deglycosylated CTR ECD WT by PNGase F for the comparison with the results of CTR ECD[N130D]. PNGase F treatment of the glycosylated CTR ECD WT decreased FITC‐sCT(22–32) affinity by sevenfold (Figs 1A and 2C) as expected from the previous report [4]. p*K*_D_ of FITC‐sCT(22–32) for CTR ECD with No PNGase F, + PNGase F, and + heat‐inactivated PNGase F was 6.25 (*K*_D_ 0.56 μm), 5.42 (*K*_D_ 3.80 μm), and 6.30 (*K*_D_ 0.50 μm), respectively. PNGase F‐catalyzed deglycosylation was confirmed with SDS/PAGE and following Coomassie blue staining (Fig. 2D).

### The ECD coincubation system forms a functional RAMP2 ECD:CTR ECD complex

**Fig. 3:**
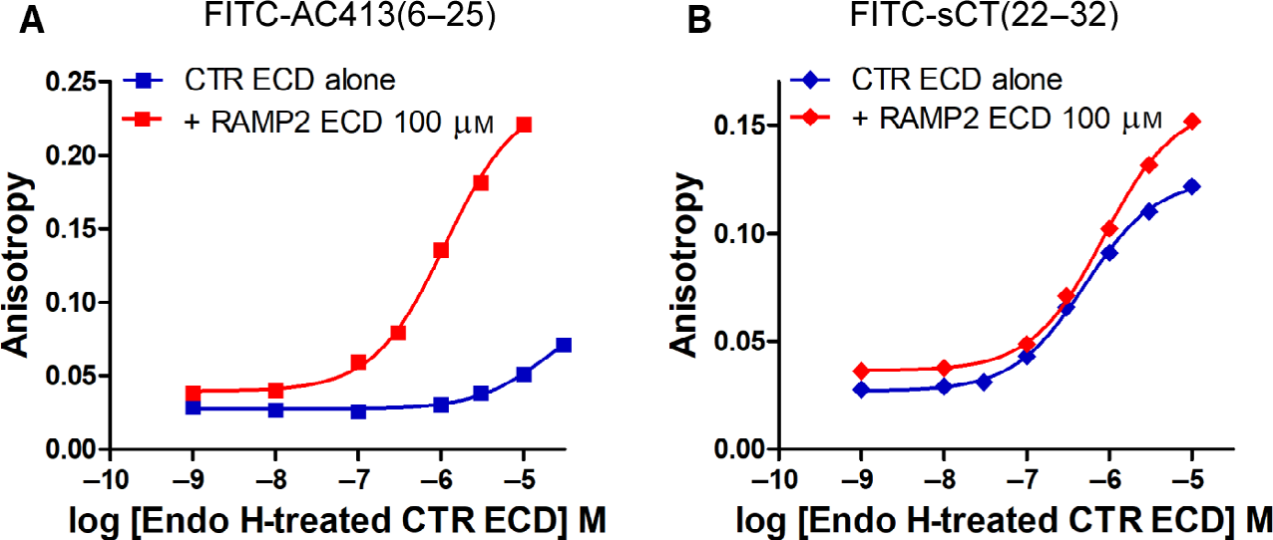
FITC‐AC413(6–25) or FITC‐sCT(22–32) binding to Endo H‐treated CTR ECD coincubated with glycan‐free RAMP2 ECD 100 μm. (A) Selective FITC‐AC413(6–25) binding to Endo H‐treated CTR ECD coincubated with RAMP2 ECD 100 μm compared with its binding to Endo H‐treated CTR ECD alone. Representative curves were shown from three independent experiments. (B) FITC‐sCT(22–32) binding to Endo H‐treated CTR ECD alone and to Endo H‐treated CTR ECD coincubated with RAMP2 ECD 100 μm. Duplicate samples per each concentration were used, and SEM was shown as error bars. When the error bars of the duplicate samples were shorter than the height of the symbol in the representative curves, they were omitted.

Amylin receptor ECD is the heterodimer complex of RAMP ECD and CTR ECD. My colleagues and I previously reported that the RAMP2 ECD and CTR ECD fusion protein (RAMP2‐CTR ECD fusion) showed enhanced amylin analog affinity compared with CTR ECD alone [8]. This suggested that the RAMP2‐CTR ECD fusion represents a functional AMY_2_ ECD. We also reported that *N*‐glycosylation of the RAMP2‐CTR ECD fusion protein enhanced an antagonistic amylin analog AC413 affinity by over 10‐fold [8]. Although the Asn‐linked GlcNAc of CTR N130 was important for the peptide affinity enhancement for CTR ECD [4, 8], whether the effect of Asn‐linked GlcNAc of CTR N130 is also applied to AMY_2_ ECD has not been reported yet. Thus, I investigated the role of Asn‐linked GlcNAc of CTR N130 in peptide binding affinity for AMY_2_ ECD. At first, I tested the formation of functional RAMP2 ECD:CTR ECD complex where CTR ECD retains Asn‐linked GlcNAc. Although tethering RAMP2 ECD and CTR ECD was shown to form functional AMY_2_ ECD [4, 8], coincubation of RAMP2 ECD and CTR ECD without a linker is also a method to enforce the RAMP2 ECD:CTR ECD complex formation. I used Endo H‐treated CTR ECD where core *N*‐glycans were trimmed to the Asn‐linked GlcNAc to investigate the role of Asn‐linked GlcNAc. I coincubated Endo H‐treated CTR ECD with excess of glycan‐free RAMP2 ECD 100 μm to enforce the formation of the RAMP2 ECD:CTR ECD complex. The ECD coincubation dramatically increased FITC‐AC413(6–25) affinity by over 20‐fold compared with Endo H‐treated CTR ECD alone (Fig. 3A and Table 1). p*K*_D_ (Mean ± SD) of FITC‐AC413(6–25) for Endo H‐treated CTR ECD alone was 4.68 ± 0.17 (*K*_D_ 21.0 ± 8.9 μm), while p*K*_D_ of FITC‐AC413(6–25) for Endo H‐treated CTR ECD with RAMP2 ECD 100 μm was 6.03 ± 0.07 (*K*_D_ 0.93 ± 0.16 μm), both from three independent experiments. These results suggest that the CTR ECD and RAMP2 ECD coincubation system represents functional AMY_2_ ECD that shows enhanced amylin analog AC413 binding over CTR ECD alone, consistent with the previous report of the RAMP2‐CTR ECD fusion protein [8]. In contrast to AC413, the affinity of FITC‐sCT(22–32) for Endo H‐treated CTR ECD was moderately decreased by twofold by RAMP2 ECD 100 μm coincubation (Fig. 3B), suggesting RAMP2 ECD addition minimally affected sCT binding. These results were consistent with the previous report that selective binding for the RAMP2‐CTR ECD fusion was observed with AC413, not with sCT [8]. p*K*_D_ of FITC‐sCT(22–32) for Endo H‐treated CTR ECD alone was 6.41 ± 0.04 (*K*_D_ 0.39 ± 0.03 μm) from three independent experiments, and p*K*_D_ of FITC‐sCT(22–32) for the RAMP2 ECD 100 μm coincubation was 6.08 (*K*_D_ 0.84 μm), respectively.

**Table 1:** Peptide ligand affinity measured in this study.

Receptor ECD	FITC‐labeled peptides	*N*	p*K*_D_ Mean ± SD	*K*_D_ (μm) Mean ± SD
Fig. 3A				
Endo H‐treated CTR ECD	FITC‐AC413(6–25)	3	4.68 ± 0.17	21.0 ± 8.9
+ RAMP2 ECD 100 μm	FITC‐AC413(6–25)	3	6.03 ± 0.07^a^	0.93 ± 0.16
Fig. 4A				
RAMP2 ECD 100 μm coincubation				
with Endo H‐treated CTR ECD WT	FITC‐AC413(6–25)	3	5.91 ± 0.08	1.24 ± 0.21
with Endo H‐treated CTR ECD N130D	FITC‐AC413(6–25)	3	5.29 ± 0.03^b^	5.13 ± 0.33
with glycan‐free CTR ECD WT	FITC‐AC413(6–25)	3	5.34 ± 0.05^b^	4.60 ± 0.53
Fig. 4B				
RAMP2 ECD 100 μm coincubation				
with Endo H‐treated CTR ECD WT	FITC‐sCT(22–32)	3	6.07 ± 0.11	0.85 ± 0.20
with glycan‐free CTR ECD WT	FITC‐sCT(22–32)	3	5.59 ± 0.16^a^	2.58 ± 0.86
Fig. 5A				
RAMP2 ECD WT‐CTR ECD fusion	FITC‐AC413(6–25)	3	6.48 ± 0.03	0.333 ± 0.026
RAMP2 ECD N130D‐CTR ECD fusion	FITC‐AC413(6–25)	3	6.47 ± 0.07^NS^	0.339 ± 0.059
Fig. 5B				
MBP‐RAMP2 ECD WT‐CLR ECD fusion	FITC‐AM(37–52) S45W/Q50W	3	7.21 ± 0.13	0.061 ± 0.021
MBP‐RAMP2 ECD N130D‐CLR ECD fusion	FITC‐AM(37–52) S45W/Q50W	3	7.34 ± 0.03^NS^	0.045 ± 0.003

^a^ *P* < 0.05 compared with the Endo H‐treated CTR ECD group. Student's *t*‐test (two‐tailed) was used for statistical analysis.

^b^ *P* < 0.05 compared with the Endo H‐treated CTR ECD WT group. ANOVA with Tukey's multiple comparison test was used for statistical analysis.

^NS^, Not significant when analyzed with Student's t‐test (two‐tailed).

### An Asn‐linked GlcNAc of CTR N130 is responsible for AC413 affinity enhancement for AMY_2_ ECD

**Fig. 4:**
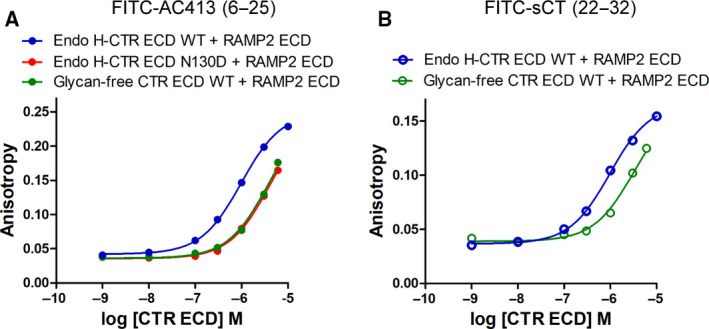
Effects of the Asn‐linked GlcNAc of CTR N130 on FITC‐AC413(6–25) or FITC‐sCT(22–32) binding for the RAMP2 ECD:CTR ECD complex. (A) FITC‐AC413(6–25) binding for Endo H‐treated CTR ECD WT or N130D and glycan‐free CTR ECD that were coincubated with glycan‐free RAMP2 ECD 100 μm. Glycan‐free CTR ECD produced from *Escherichia coli* was used for the formation of the RAMP2 ECD:CTR ECD complex without any *N*‐glycans. Representative curves were shown from three independent experiments. (B) FITC‐sCT(22–32) binding for Endo H‐treated CTR ECD WT and glycan‐free CTR ECD that were coincubated with glycan‐free RAMP2 ECD 100 μm. Representative curves were shown from three independent experiments. Duplicate samples per each concentration were used, and SEM was shown as error bars. When the error bars of the duplicate samples were shorter than the height of the symbol in the representative curves, they were omitted.

I purified Endo H‐treated CTR ECD[N130D] where the Asn‐linked GlcNAc at N130 was absent and also purified glycan‐free CTR ECD produced from *E. coli* as previously reported [8]. I used these CTR ECDs for the coincubation with glycan‐free RAMP2 ECD and measured FITC‐AC413(6–25) affinity for the RAMP2 ECD:CTR ECD complex. Interestingly, the lack of the Asn‐linked GlcNAc of CTR N130 by the N130D mutation decreased FITC‐AC413(6–25) affinity by fourfold and the similar affinity decrease was observed when glycan‐free CTR ECD was used for the coincubation with RAMP2 ECD (Figs 1B and 4A and Table 1). p*K*_D_ (Mean ± SD) of FITC‐AC413(6–25) for the RAMP2 ECD coincubation with Endo H‐treated CTR ECD WT, Endo H‐treated CTR ECD[N130D], and the glycan‐free CTR ECD was 5.91 ± 0.08 (*K*_D_ 1.24 ± 0.21 μm), 5.29 ± 0.03 (*K*_D_ 5.13 ± 0.33 μm), and 5.34 ± 0.05 (*K*_D_ 4.60 ± 0.53 μm), respectively, from three independent experiments. These suggest that the Asn‐linked GlcNAc of CTR N130 is responsible for the *N*‐glycosylation effect on AC413 affinity enhancement for AMY_2_ ECD. ANOVA with Tukey's *post hoc* test was performed with p*K*_D_ of FITC‐AC413(6–25), and p*K*_D_ of FITC‐AC413(6–25) of the Endo H‐treated CTR ECD WT group was significantly different from that of the Endo H‐treated CTR ECD[N130D] group and the glycan‐free CTR ECD WT group (Fig. 4A and Table 1).

Peptide hormone sCT is known as a high affinity agonist both for CTR and for AMY [18]. Sequence alignment of amylin, AC413, sCT, and related others is shown in Fig. 1D. I found that the RAMP2 ECD coincubation with glycan‐free CTR ECD decreased FITC‐sCT(22–32) affinity by threefold compared with RAMP2 ECD coincubation with Endo H‐treated CTR ECD (Figs 1B and 4B and Table 1). p*K*_D_ (Mean ± SD) of FITC‐sCT(22–32) for the RAMP2 ECD coincubation with Endo H‐treated CTR ECD and glycan‐free CTR ECD was 6.07 ± 0.11 (*K*_D_ 0.85 ± 0.20 μm) and 5.59 ± 0.16 (*K*_D_ 2.58 ± 0.86 μm), respectively, from three independent experiments. This difference was statistically significant when analyzed with Student's *t*‐test (*P* < 0.05). These results are consistent with Fig. 4A, suggesting that the several different amino acids between AC413 and sCT did not affect their enhanced affinity for glycosylated AMY_2_ ECD.

### *N*‐glycosylation effects of RAMP2 N130 on peptide ligand affinity for RAMP2‐CTR ECD fusion or RAMP2‐CLR ECD fusion proteins

**Fig. 5:**
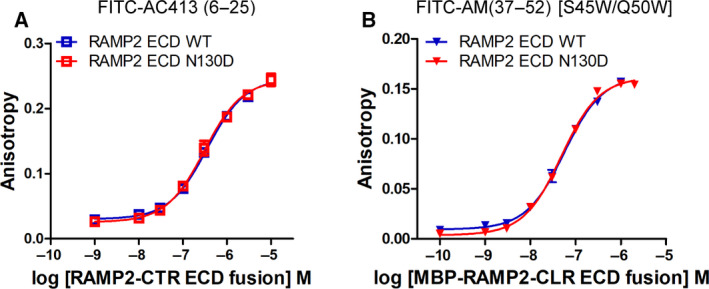
Effects of the RAMP2 N130D mutation on peptide ligand binding for RAMP2‐CTR ECD fusion or RAMP2‐CLR ECD fusion proteins. (A) FITC‐AC413(6–25) binding to RAMP2 WT or N130D‐CTR ECD fusion protein. Representative curves were shown from three independent experiments. (B) FITC‐AM(37–52)[S45W/Q50W] binding for MBP‐tagged RAMP2 WT or N130D‐CLR ECD fusion protein. Representative curves were shown from three independent experiments. Duplicate samples per each concentration were used, and SEM was shown as error bars. When the error bars of the duplicate samples were shorter than the height of the symbol in the representative curves, they were omitted.

RAMP2 ECD has one *N*‐glycosylation site, N130. Since I used glycan‐free RAMP2 ECD produced from *E. coli*, the RAMP2 ECD coincubation system with CTR ECD does not address any possible *N*‐glycosylation effect of the RAMP2 N130. I investigated the effect of RAMP2 N130 *N*‐glycosylation on peptide ligand affinity. RAMP2 WT or N130D‐CTR ECD fusion proteins were expressed and purified. I found that RAMP2 with the N130D mutation did not alter the FITC‐AC413(6–25) binding affinity (Figs 1C and 5A and Table 1). p*K*_D_ (Mean ± SD) of FITC‐AC413(6–25) for the RAMP2 WT‐CTR ECD fusion and RAMP2 N130D‐CTR ECD fusion proteins was 6.48 ± 0.03 (*K*_D_ 333 ± 26 nm) and 6.47 ± 0.07 (*K*_D_ 339 ± 59 nm) from three independent experiments. MBP‐tagged RAMP2 WT or N130D‐CLR ECD fusion proteins were also purified, and the affinity of FITC‐AM(37–52)[S45W/Q50W] was measured. Consistent with the results of RAMP2‐CTR ECD fusion protein, the affinity of FITC‐AM(37–52)[S45W/Q50W] for the MBP‐RAMP2‐CLR ECD fusion protein was not altered by the RAMP2 N130D mutation (Fig. 5B and Table 1). p*K*_D_ (Mean ± SD) of FITC‐AM(37–52)[S45W/Q50W] for the MBP‐RAMP2 WT‐CLR ECD fusion and MBP‐RAMP2 N130D‐CLR ECD fusion proteins was 7.21 ± 0.13 (*K*_D_ 61 ± 21 nm) and 7.34 ± 0.03 (*K*_D_ 45 ± 3 nm), respectively, from three independent experiments. These results suggest that RAMP2 *N*‐glycosylation is dispensable for peptide ligand affinity both for RAMP2‐CTR ECD fusion and for RAMP2‐CLR ECD fusion proteins.

## Discussion

I report for the first time to my knowledge that the Asn‐linked GlcNAc of CTR N130 is a significant affinity enhancer for AMY_2_ ECD. The previous report suggested that CTR ECD N130 is the most responsible *N*‐glycosite for peptide affinity enhancement for CTR ECD and that the Asn‐linked GlcNAc of N130 is sufficient for this effect [4]. A following report showed that *N*‐glycosylation enhances peptide ligand affinity for AMY_1/2_ ECD and that CTR N130 is largely responsible for the affinity enhancement at least for AMY_1_ ECD [8]. Here, I show that the CTR ECD N130 is also responsible for peptide affinity enhancement for AMY_2_ ECD and that the Asn‐linked GlcNAc of CTR ECD N130 plays an important role in enhancing peptide binding for AMY_2_ ECD.

My colleagues and I previously suggested a working mechanism of how the Asn‐linked GlcNAc of CTR N130 enhances peptide hormone affinity for CTR ECD [8]. Unexpectedly, the crystal structures of Endo H‐treated/Asn‐linked GlcNAc‐containing CTR ECD were almost identical to the structure of glycan‐free CTR ECD with a subtle structural difference. Direct interaction between the Asn‐linked GlcNAc and peptide hormone sCT was not found in the crystal structures ruling out the hypothesis of direct interaction between them. We also examined whether CTR ECD residues near the Asn‐linked GlcNAc of N130 mediate the *N*‐glycosylation effect. However, we could not find any evidence for CTR ECD residues that mediate the *N*‐glycosylation effect on the peptide affinity enhancement from our mutagenesis experiments. We found that the Asn‐linked GlcNAc of CTR N130 increased the association rate of sCT by threefold and decreased its dissociation rate by twofold resulting in sixfold affinity enhancement. In addition, the Asn‐linked GlcNAc of CTR N130 affected the dynamics of CTR ECD by stabilizing two β‐strands and by increasing flexibility of a turret loop located in the peptide binding pocket. Our results of molecular dynamics simulations and hydrogen–deuterium exchange mass spectrometry suggested that the Asn‐linked GlcNAc of CTR N130 controls receptor dynamics to enhance peptide hormone calcitonin affinity. These results are also consistent with the previous work that the Asn‐linked GlcNAc is entirely responsible for acceleration of protein folding and accounts for two thirds of the native state stabilization of the whole *N*‐glycans [19].

Other research groups reported that *N*‐glycosylation affects receptor dynamics with a subtle structural change [20, 21]. Xin and Radivojac analyzed Protein Data Bank and found that only 7% of glycosylated proteins undergo global changes in their structures with > 2 Å of root‐mean‐square deviation [20]. Consistently, Lee *et al*. analyzed crystal structures of glycosylated proteins and their glycan‐free forms and found that *N*‐glycosylation does not induce significant changes in protein structure. However, when they performed molecular dynamics simulations they found that *N*‐glycosylation decreased dynamics of a subset of proteins that can lead to an increase in protein stability [21].

The mechanisms of RAMP ECD actions at CLR or CTR ECD for selective peptide hormone binding have been suggested as a combination of direct interaction with peptide ligands and allosteric effects on receptor conformation [22, 23, 24, 25, 26]. Crystal and recent cryo‐EM structures indicate that the α1 helical domain of CLR ECD interacts with RAMP1/2 ECD as previously described [22, 27, 28]. Similarly, RAMP1 ECD was predicted to interact with the α1 helical region of CTR ECD and the mutagenesis results of the predicted interface between RAMP1 ECD and CTR ECD support this idea [26]. In addition, molecular dynamics simulations indicated that the presence of RAMP1 ECD reduced the fluctuations of CTR ECD α1 helical domain [26]. Since the α1 helical domain is away from the peptide binding pocket, these are consistent with the idea that RAMP ECD allosterically modulates CTR or CLR ECD conformation for selective peptide hormone binding. The findings of the current study suggest that the potential allosteric effects of RAMP2 ECD on CTR ECD did not alter the *N*‐glycosylation effect of CTR ECD on peptide affinity enhancement since peptide affinity enhancement by the Asn‐linked GlcNAc of CTR N130 also remained effective for the RAMP2 ECD:CTR ECD complex. In addition, sCT affinity for AMY_2_ ECD was also increased by the Asn‐linked GlcNAc of CTR N130, suggesting that the *N*‐glycosylation effect of AMY_2_ ECD is likely to be independent of amino acid sequences of peptide ligands.

Maximal anisotropy values of FITC‐sCT(22–32) with Endo H‐treated CTR ECD appeared smaller than that of FITC‐sCT(22–32) with RAMP2 ECD coincubation (Fig. 3B). Maximal anisotropy values are largely determined by the size of the receptor bound with an FITC‐labeled probe. The tumbling rate of the FITC probe is slowed by the increased size of the receptor–probe complex resulting in the increased anisotropy values of the FITC probe. RAMP2 ECD coincubation with CTR ECD produces the RAMP2 ECD association with CTR ECD, and this RAMP2 ECD:CTR ECD complex is apparently greater than CTR ECD alone. Accordingly, the maximal anisotropy of FITC‐sCT(22–32) was increased with RAMP2 coincubation in Fig. 3B. The increased anisotropy of the FITC probe was also shown in our recent report that used RAMP2 ECD coincubation or RAMP2 ECD fusion to CTR ECD [29].

RAMP2 ECD has one potential *N*‐glycosylation site at N130, and it was dispensable for peptide hormone affinity (Fig. 5). These results are consistent with the idea that RAMP2 ECD N130 is located away from the peptide binding pocket of the RAMP2 ECD:CTR ECD complex (Fig. 1C). While RAMP1 ECD does not have any potential *N*‐glycosylation site, RAMP3 ECD has four potential *N*‐glycosylation sites. The *N*‐glycosylation effect of AMY_3_ ECD (RAMP3 ECD:CTR ECD complex) will also be of interest for the future studies.

This study reports that the Asn‐linked GlcNAc of CTR N130 enhances peptide hormone affinity for AMY_2_ ECD. A previous study showed that the CTR N130 *N*‐glycosylation site is conserved in other class B GPCRs such as corticotropin‐releasing hormone receptors [8]. Although there is a case that receptor *N*‐glycosylation is dispensable for class B GPCR functions [30], future studies for *N*‐glycosylation and Asn‐linked GlcNAc of other peptide hormone receptors will expand our understanding of how *N*‐glycosylation affects class B GPCR function.

## Conflict of interests

The author declares no conflict of interest.

## Author contributions

SL conceived, designed, and performed experiments and analyzed data. SL wrote the manuscript.

## Data Availability

Data will be available from the corresponding author upon reasonable request.
